# Computer Vision in Dairy Farm Management: A Literature Review of Current Applications and Future Perspectives

**DOI:** 10.3390/ani15172508

**Published:** 2025-08-26

**Authors:** Veronica Antognoli, Livia Presutti, Marco Bovo, Daniele Torreggiani, Patrizia Tassinari

**Affiliations:** Department of Agricultural and Food Sciences (DISTAL), University of Bologna, 40127 Bologna, Italy; veronica.antognoli2@unibo.it (V.A.); livia.presutti2@unibo.it (L.P.); daniele.torreggiani@unibo.it (D.T.); patrizia.tassinari@unibo.it (P.T.)

**Keywords:** cow, PLF, deep learning, heat stress, machine vision

## Abstract

Computer vision is rapidly transforming the field of dairy farm management by enabling automated, non-invasive monitoring of animal health, behavior, and productivity. This review provides a comprehensive overview of recent applications of computer vision in dairy farming management operations, including cattle identification and tracking, and consequently the assessment of feeding and rumination behavior, body condition score, lameness and lying behavior, mastitis and milk yield, and social behavior and oestrus. By synthesizing findings from recent studies, we highlight how computer vision systems contribute to improving animal welfare and enhancing productivity and reproductive performance. The paper also discusses current technological limitations, such as variability in environmental conditions and data integration challenges, as well as opportunities for future development, particularly through the integration of artificial intelligence and machine learning. This review aims to guide researchers and practitioners toward more effective adoption of vision-based technologies in precision livestock farming.

## Simple Summary

Computer vision is becoming a transformative tool in dairy farm management, offering farmers a more efficient, accurate, and non-invasive way to monitor herd health, behavior, and productivity. This enables real-time decision-making, reduces labor costs, and minimizes errors associated with manual observation. Additionally, computer vision systems can enhance animal welfare by identifying stress or discomfort early, leading to timely interventions that improve overall herd well-being and productivity. When integrated with other smart farming technologies, such as automated milking systems and precision feeding, computer vision contributes to sustainable and data-driven farm management, optimizing milk yield while reducing waste and resource use. Ultimately, the adoption of computer vision in dairy farming supports both economic efficiency and animal welfare, aligning with the growing demand for sustainable agricultural practices.

## 1. Introduction

The growing demand for animal-derived products called for an expansion of the livestock sector. With regard to the dairy sector, the demand for milk will increase exponentially by 2050 [1], leading to the need for improved farm-management procedures. Traditional farming relies on farmers’ observations and experience; however, that alone is not sufficient to ensure the feasibility of livestock farming on a large scale. Therefore, many precision livestock farming (PLF) technologies have been spreading worldwide as very efficient tools that support the farmers in the decision-making process by providing them with valuable information on animal welfare, health, production, and reproduction [2] through the automated monitoring of the herd [3].

The most widely used methods to identify, track, or measure animal-based parameters on cattle are wearable devices. However, their effectiveness is affected by the fact that they can be lost, damaged, or cause stress to the animal [4,5,6], implying discomfort for the cows and economic losses for the farmers. On the contrary, the presence of cameras does not impact the animals, thereby preventing any stress associated with their use. Indeed, the computer vision approach represents a cost-effective alternative to wearable devices, which can provide a large amount of animal-based information, replacing the farmer in many repetitive and time-consuming tasks [7,8]. However, it must be considered that data obtained by wearable devices often present availability limitations due to software permissions, leading to a more difficult data incorporation between different technologies [9]. A more comprehensive approach, helpful to consider different data derivations, is essential for the improvement of livestock management and farm sustainability. In this context, computer vision plays a key role as an enabling technology for PLF, allowing automated, non-invasive, and continuous monitoring of animals and helping farmers make more informed decisions based on data.

### 1.1. Common Stages in Computer Vision Methodologies

The widespread deployment of sensors for routine livestock monitoring led to a significant enhancement in both the volume and the quality of data collected on farms, allowing a more comprehensive and robust application of statistical analysis and numerical modelling techniques. In this context, researchers throughout the last decade focused on the development of computer vision systems with a combination of machine learning models, aiming at the improvement of data analysis. For instance, You Only Look Once (YOLO) [10] models are a group of convolutional neural networks (CNNs) used for object detection and image classification that are vastly applied because they are developed to be fast and accurate and easy to implement; in fact, their structure allows them to process the whole image in one single step. They are mentioned as a group because they are continuously re-modelled and re-sized to better fit different needs [8]. YOLO is a popular object-detection model, known for its real-time performance and accuracy, that has been used in several studies on dairy cow monitoring. Different versions of YOLO have been developed to improve its efficiency. Similarly, CNNs represent a widely adopted family of deep learning methods, extensively developed and adapted to various contexts, and integrated into neural network architectures with diverse characteristics. CNNs are particularly well-suited for agricultural applications because they excel at handling complex and varied image data, such as crops, soils, and livestock captured under different lighting, weather, and growth conditions. Their ability to automatically extract and learn hierarchical features makes them robust in distinguishing subtle differences, for example, between healthy and diseased animals. In addition, CNNs are highly efficient for real-time image processing, which is critical for applications like precision farming, where timely decisions directly impact yield and resource management. These models can be combined with numerous algorithms, such as DeepSORT for multi-object tracking [11], the WhenToRead module to increase ear-tag recognition [12], and CenterNet for keypoint detection [13], among others, to construct frameworks adapted to specific requirements. The procedure adopted in most of the reviewed articles comprised the following steps:

(a) Data collection

The data collection phase follows two different approaches depending on the choice of dataset for the image or video analysis. Specifically, some studies relied on pre-recorded datasets that were already available for processing [14]. In contrast, others opted to create their own datasets using RGB cameras [7,8,15], depth cameras [9,16], infrared cameras [17,18], and thermal cameras [5,13,19], selected and positioned according to the specific objectives of the research.

(b) Data pre-processing and processing

In this phase, frames were annotated using bounding boxes [20], keypoints, or point clouds [21], depending on the annotation methodology selected by the authors according to the objective of the study, in preparation for subsequent processing. This procedure has been carried out either manually or supported by automated methods, such as 3D pose estimation software [22], Labelme [23], LabelImg [24], or Computer Vision Annotation Tool (CVAT) [7]. The extracted frames are elaborated through, for instance, background removal [21], image enhancement [24], signal frequency selection [25], and finally data augmentation techniques to improve the overall performance of the framework [11,26,27] and remove obstructions, noise, interferences, or any other factors that may interfere with or degrade the performance of machine learning algorithm analyses and improve feature classification.

(c) Data analysis

The final steps of this process involve the object detection and classification, performed by several different models, including the You Only Look Once (YOLO) [10] and R-CNN [28] families.

(d) Results assessment

This phase includes the metrics utilized to determine the performance of the system, such as intersection-over-union (IoU) [29] and average IoU to assess the degree of similarity between two images, precision, and recall that are combined in the balanced F1-score, confidence score, average precision, and mean average precision.

**Figure 1:**
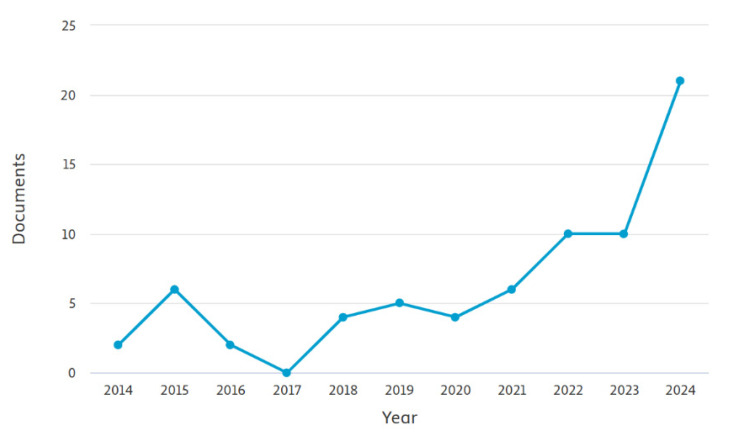
Trend of the bibliographic sources investigating computer vision applications related to livestock management.

In the last 10 years, computer vision and deep learning methods have experienced important growth in terms of technological development, bearing the potential to revolutionize livestock farming management. In agreement, Figure 1 shows the trend of bibliographic sources investigating computer vision applications related to livestock management resulting from a search through Scopus by filtering title, abstract, and keywords.

To reduce the amount of time spent on repetitive activities related to herd monitoring, a reliable instrument that provides decisional support is strictly necessary. Computer vision systems have found a promising application in several aspects related to farm management since they can be applied to different settings to obtain precise, accurate, and objective data. By automating time-consuming tasks and enabling an objective processing that provides decision-making support, machine learning technologies offer significant assistance to livestock farmers. In the context of increasingly large-scale herd management, the adoption of such technologies is crucial for minimizing human errors and operational costs while simultaneously enhancing the efficiency of herd management practices, such as reproduction, nutrition, and production processes.

### 1.2. Aim and Scope of This Review

The following paper presents the findings of a literature review conducted to identify and summarize the key potential applications of computer vision systems to dairy cattle management. The main objective of this review is to investigate and organize the existing literature in this field of research to develop a more comprehensive understanding of the topic. The remaining sections of this document are organized as follows: The second section outlines the methodology of this review and presents the collection of papers analysed in this study. The paragraphs from third to ninth will present, in the subsequent order, the applications of computer vision and machine learning technologies for identification and tracking, feeding and rumination, body condition score (BCS), respiration rate and heat stress, lameness and lying behavior, mastitis and milk yield, and social behavior and oestrus of dairy cattle.

## 2. Review Methodology

### 2.1. Criteria for the Source Selection

**Figure 2:**
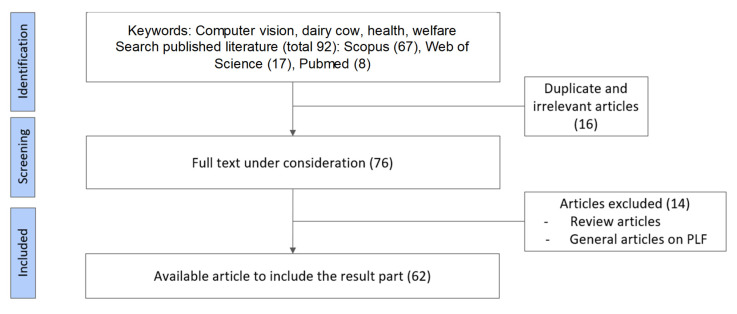
Flow diagram of the bibliographic source selection process.

The bibliographic research was conducted by consulting Scopus, Web of Science, and PubMed databases, selecting the sources with titles, abstracts, and keywords. The combination of search terms used on Scopus included TITLE-ABS-KEY ((dairy cow OR cattle OR cow) AND (computer vision OR machine vision OR vision system) AND (management OR tracking OR identification OR feeding)) AND (LIMIT-TO (LANGUAGE, “English”)). Whereas, on the other databases, the research was carried out leveraging the keywords “dairy cow”, “computer vision”, “machine learning”, and “management”. The limitations that were applied concern time and language. Regarding the time limitation, the selected papers range between 2014 and 2024, when the bibliographic search ended. Meanwhile, texts from book chapters, peer-reviewed articles, and conference abstracts written in English were considered for a full text review of the document. At the end of this screening, a database of 92 documents was obtained. Thereafter, articles that did not align with this review’s scope were excluded. Figure 2 outlines the essential phases of the selection process.

### 2.2. Review of the Reviews: Progresses, Overlaps, and Research Trends

The literature reviews published in recent years tend to focus on a single application of computer vision, such as body weight estimation [30], behavior recognition [31,32], or animal identification [33], thus lacking a broader overview of its potential applications and the current state of the art across the various specific domains. Otherwise, Refs. [34,35] propose a more comprehensive analysis of articles that apply computer vision and machine learning, focusing on giving a technical outline of the frameworks’ characteristics. With this review, we aim to provide a comprehensive summary and general overview of the potential applications of computer vision within the farm setting as a decision-support tool for farmers, with the goal of optimizing farm-management practices.

## 3. Identification and Tracking

The ability to identify and track the single animal is fundamental for optimal livestock farming management. In the dairy sector, providing each animal with an identification enables the traceability of the animal’s products to guarantee the consumers’ safety. Additionally, tracking and analysing animals’ behavior is of great importance; in fact, it can help the farmer in the detection of animals that need intervention or health care [36]. For this purpose, different types of precision livestock farming (PLF) devices are already made available on the market, equipped with the ability to recognize the individual and track its movements inside the barn. The vast majority of them are wearable devices; thus, they need physical contact with the animal to be effective and, as a result, can be stressful for the animals. Furthermore, most of these technologies are commonly lost or destroyed by the animal’s physical interactions with them [7], causing economic losses to the farmer. One of the mainly used PLF technologies for livestock identification and tracking is the Radio-Frequency Identification (RFID) technology, which wirelessly transmits recorded information. Nevertheless, those technologies can incur communication failure, both due to the sensors’ loss or connection problems between the sensor and the farmer’s computer [16]. The aim of PLF is to provide a real-time technology that can continuously monitor animals in the barn [37]. Therefore, modern solutions applied in the dairy industry ask for a systematic, reliable, and accurate system that can recognize and identify each animal automatically in order to support the farmer in managing the herd in terms of health, behavior, production, and welfare of the cows and, additionally, in order to improve the traceability of the animals’ products over time. An efficient method for this is through the use of computer vision or by combining information provided by other technologies with that provided by computer vision. This technology offers a valid alternative to wearable sensors and a non-contact solution that is also cost-effective [14,16].

Animal biometric identification is a strong challenge for researchers all over the world. The authors are trying different strategies to cope with the fact that real-time recorded images are not always of an ideal quality and consequently can cause problems in the development of software that can generalize the images from the background, maintaining a high percentage of accuracy [14]. In the analysed papers, 80% of the authors decided to use an RGB (red, green, blue) camera, and the rest have leveraged instead infrared technology or a combination of the two [5]. Most of the articles decided to focus solely on identification; however, 30% of them expanded the use of the camera to track the cows inside the barn and to monitor usual behaviours such as drinking and feeding. More than 50% of the models presented by the analysed authors focused on the whole body of the animal, with the aim of recognizing the individual based on the coat pattern. While 30% of the articles identify subjects focusing on biometric characteristics of the face or particularly of the muzzle. Ref. [38] focuses on the analysis of osseous and cartilaginous bovine faces’ components, leveraging Euclidean Distance Matrix Analysis and vector projection techniques combined with machine learning techniques. The indicated anatomical region has been selected for the analysis since it concentrates in a relatively small space different geometrically complex morphological patterns that can be recognized by algorithms. It must be considered that alternatives to biometric features exist when it comes to recognition and identification. For instance, Ref. [8] has researched algorithms specialized in reading different types of fonts and handwriting to recognize numbers written on ear tags. A useful algorithm to obtain data with this method is Scene Text Recognition (STR), which is a type of Optical Character Recognition (OCR) specialized in recognizing text extracted from natural images.

Furthermore, processing speed is of great importance for real-time detection of animals in large dairy farms, where a great computational capacity is required for the model. To overcome this problem, the previously mentioned authors introduced the “WhenToRead” module so that instead of utilizing only the information provided by one image, the model can make better predictions relying on video-based recognition. With this method, previous frames are compared every time a new frame is analysed, and only the most informative one is kept. This helps the model make better predictions while using less computing power, making it easier to apply in real-world situations.

In order to assign an ID and then track the animals, the system has to comprehend two algorithms: one designated to the detection of the individual, which can be both image- or video-based, and another that performs the recognition of the animal. Researchers attempted to focus on different biometric features to implement animal detection and recognition, leading to the development of specific algorithms able to identify cows through the analysis of coat pattern [26] and morphologic features of the whole body of the cow [18,39] or limitedly of the side [5], back [14], face, and muzzle [7,15,16,40]. Likewise, utilizing an RGB-D camera that provides information both on colour and depth of the image, it is possible to extract individual 3D cow models and identify animals through their gait [41]. The majority of the authors detected animals leveraging YOLO models, providing extracted features that consequently entered a multi-object tracking layer that assigns the identification number to the individual throughout the video stream, achieving cow recognition [8]. Bergman et al. [15] firstly made a comparison of different detection and recognition algorithms, discovering that the best-performing algorithm consisted of a combination of YOLOv5 for facial detection (obtaining a mean average precision mAP = 97.8%) and a Vision-Transformer model inspired by human facial recognition (with classification accuracy = 96.3%) with a classification speed of 20 milliseconds per frame. This combination of algorithms was selected to obtain real-time efficiency, allowing the model to detect and recognize every cow in the same batch at the same time. As the authors stated, better results could be achieved by enlarging the training dataset for the Transformer model, which can consequently be more precise. One of the greatest challenges for this technological field consists in the fact that when the images or the videos are recorded, the algorithms have great difficulties in focusing and extracting useful features from images that have different quality and backgrounds. Many authors focused on the creation of a model that could be applied efficiently, not only limited to a specific context but also capable of performing background generalization to maintain high recognition accuracy in different settings. Ref. [14] developed the BottleNet Transformer, fusing the architecture of a CNN and a Transformer to extract both local and global features with one algorithm that was adapted to the real scenario of large-scale farms by adding both Counterfactual Attention Learning and Graph Sampling to the network.

Another combination strategy attempted consisted in the development of a deep learning-based individual recognition model that combines Mask R-CNN instance segmentation and the ResNet101 image classification model to identify individual cows in milking parlours. This model obtained an accuracy of 97.58% with ResNet101 and an average accuracy of 96.73% with the Mask R-CNN model, outperforming other combinations of Mask R-CNN with other individual classification networks, such as VGG16, ResNet34, and GoogleNet [42].

Individual identification of animals can find an application not only in real-time monitoring of the herd but also in animal production traceability. In fact, Ref. [17] used Siamese networks for the recognition and identification of dairy cattle in different stages of life: in this case, the authors applied the model to recognize young subjects that were previously identified after a year of growth, obtaining an F1-score of 73%.

As a matter of fact, calves are particularly susceptible to rapid growth; hence, identification is an issue to perform and can affect the predictive performance of the algorithms. It has been demonstrated by [21] that algorithms that perform identification of dairy calves based on 3D images of the dorsal area are robust enough to remain accurate through the growing stage, as they are able to extract unrepeatable biometric features that are not affected by the physical changes typical of this period.

## 4. Feeding and Rumination Behavior

Monitoring the feeding behavior and rumination activity of dairy cows supports experts in the detection of abnormalities of the health and welfare status of animals. It has to be considered that animals’ nutrition and digestive processes are directly related to their productivity and quality of production [43]. For instance, dairy cows experiencing heat stress register a decrease in feed intake and rumination, leading to a compromised production of milk [44]. Real-time monitoring could be performed by image processing algorithms combined with machine learning models. This technology enables tracking of each cow’s movements and behaviours throughout the farm [45]. Ref. [43] utilized the YOLOv8 object detection framework, with a decoupled head that performs separate classification and regression to increase possible accuracy in terms of identification and tracking, individuating a limit for the system in the resolution of the cameras that could be improved to obtain a mAP higher than 50.2%. Ref. [46], instead, employed the YOLOv3 model, trained with manually labelled images, to efficiently predict feeding behavior. The authors decided to analyse the number of visits, the mean visit duration, the mean interval between visits, and the total feeding time, considering as a new visit the moment in which the cow inserted the head through the feed rail. The research group stated that increasing the interval of image acquisition helps the model in the prediction, as they obtained an overall accuracy of 99.4%. It has emerged from the analysed articles that to increase model performance, it is appropriate to train the models with numerous and dissimilar data rather than to train them with homogeneous data [47]. Multiple wearable devices have been used to monitor rumination activity in dairy cattle, such as microphones [48], nosebands [49], and accelerometers [50]; however, as devices that must be in contact with the animal to function properly, they can be lost or broken. Neural networks and machine learning could be a viable solution to assess animal behavior, utilizing non-contact devices [27]. Ref. [27] presented a computer vision model that combines VGG16 and ResNet to recognize when the cow is chewing or swallowing, obtaining an accuracy of 98.12%, resulting in an efficient system for monitoring ruminal activity. Authors concentrate on observing rumination and chewing parameters, since they are strictly related to animal health and welfare. However, alterations in the rumination pattern are not all symptoms of illness; they instead could be physiological manifestations related to productivity, general physiological state, and feed administration [51,52,53]. Monitoring these activities can support the farmers to recognize sick animals and to select more efficient feeding ratios or management strategies that better suit the needs of the herd.

## 5. Body Condition Score Assessment

Computer vision systems enable farmers to rely on real-time monitoring of the animals’ body condition score (BCS), allowing them to dispose of a large amount of information that is directly related to health status, feed suitability, animal production, and growth. BCS is itself an instrument that aims to evaluate animals’ energy reserves (Spoliansky et al., 2016) (Edmonson et al., 1989) [54,55]. In addition, the use of machine learning algorithms enables an immediate recognition of the onset of physiological abnormalities throughout the herd, such as metabolic issues or decreased productivity. Furthermore, real-time automatic monitoring of animals supports the farmer in identifying the extent of the problem—for instance, if the herd is almost totally affected by the disease or if only some individuals are involved.

Nowadays, the most used systems to measure body weight are walk-over weighing systems or manual observations, methods that are neither time nor cost efficient, especially in large-scale contexts, and require a trained observer who can assign the BCS [56].

In this regard, the use of low-cost three-dimensional (3D) cameras with a depth sensor has been proposed by all the authors; they are summarized in Table 1. The cameras have been applied, combined with different machine learning algorithms, to find a solution that could improve the efficacy of body weight prediction and of a more general morphometric analysis. In fact, 3D depth cameras provide images that allow tracking not only the object contours but also its structure, providing three-dimensional information about the animals [57]. Ref. [55] proposed a three-dimensional algorithm, developed in the Matlab (The MathWorks Inc., Natick, MA, USA, v2016) environment, that provides a topographical overview of the animal’s body. Extracting fourteen features from the video of each individual cow, this algorithm can predict the BCS, with a coefficient of determination of 0.75. Ref. [57] observed that the BCS could be estimated utilizing the cows’ back profiles, since they change along with body condition. The authors compared each cow’s back profile, extracted from the videos, with a fitted polynomial surface to assess the deviation degree between them, utilizing a 3D-BCS model that obtained a coefficient of determination of 0.70. Ref. [56], instead, has succeeded in obtaining a coefficient of determination of 0.98, utilizing a Mask R-CNN approach integrated with a linear mixed model in the forecasting cross validation. The authors found that R-CNN has great effectiveness in segmenting cows’ body images from the background and returned the best goodness of fit compared to single-thresholding and adaptive thresholding methods. The evaluation of the body condition can be part of a more holistic approach, hence integrated with other information from different sources, such as wearable devices [58,59], to achieve a more comprehensive overview of the individual animal and of possible management improvements that can be completed to increase the health and welfare of the herd. Ref. [9] proposed a cloud computing technology based on a cooperative learning approach that relies on both early and late fusion methods to align predictions obtained with different predictive models. This method allows for overcoming technical difficulties related to on-farm data accessibility and integration with the aim of obtaining as output a phenotype prediction. The accuracy obtained related only to the BCS evaluation performed with ResNet-50 and was 96.2%.

## 6. Respiration Rate and Heat Stress

Respiration rate is a parameter commonly employed for the identification of animals affected by respiratory diseases or heat stress. In the latter, it must be considered that thermoregulation allows animals to keep in balance energy dedicated to heat dissipation and production: if the environmental conditions prevent said balance, animals are forced to live outside their Thermo-Neutral Zone (TNZ). This imbalance results in compromised physiological activities and a reduction in milk yield and quality [23]. Production loss impacts negatively on the farm’s finances; hence, milk yield monitoring has been the parameter for heat stress recognition for many years. Actually, the effects on animals’ productivity are the consequence of a series of physiological alterations, such as reduction in feed intake, increased respiration and heart rates, and altered lying behavior [44]. Respiration rate (RR) is a particularly useful parameter to monitor with reference to heat stress; in fact, its onset is immediately encountered when the thermal conditions get out of the TNZ; therefore, it can give the farm’s managers useful information on how to activate or optimize cooling systems [25]. For years, the evaluation of RR has been carried out visually by a trained observer, resulting in a time-consuming activity that requires specific training [60]. In consideration of the necessity to find an alternative for the monitoring of RR in livestock, many authors proposed the use of wearable sensors [61], such as MP3 recorders [62], pressure sensors [63], and belts to monitor flank movements [64]. With regard to the type of camera to be used for this purpose, infrared thermal imaging has been proposed but presents some limits: the measurements can be affected by environmental temperatures, and the animal’s head has to be in a certain position to obtain useful images, sometimes recurring to a restraint that can cause stress to the animal [65]. In addition, the infrared thermal cameras’ cost is higher than that of RGB cameras [25]. The camera position most favoured by the researchers is located above the cubicles or the stalls: this choice of device positioning is linked to the fact that RR recognition in standing cows is very difficult to perform due to interfering movements and slight breathing movements [23]. Indeed, RR can be computed by focusing on the intensity variation of pixels in some defined regions of interest (ROI) that, in this case, are cows’ abdomens or chests [23].

Refs. [23,25] have both performed cows’ and ROI recognition by YOLOv8- and YOLOv5-based networks, respectively, and subsequently applied Fast Fourier Transform (FFT) to extract RR from data frames. The FFT is an algorithm that has already found application in human research [66] and has proven to be suitable for this goal.

Ref. [67] stated that the resting state could give us more reliable information on animals’ health since this state is less affected by movement disturbances because the cow is not walking, drinking, or interacting with other cows. In fact, the authors decided to employ firstly the YOLACT (You Only Look at CoefficienTs) as a deep learning algorithm that recognizes and segments the cows from the background; secondly, a VGG16 fused with a Bi-LSTM was used to recognize the cow’s resting behavior with a precision over 0.95 (i.e., resting while standing, resting while lying); and finally, RR was computed using optical flow methods. Optical flow is the change in the movement of the pixels in an image and can be related to the object of the image or to the device used for the recording: when the cow is breathing, the body moves horizontally and rhythmically, causing a reciprocal movement in the direction of the optical flow in the recorded images. A powerful tool in this regard is the Lucas–Kanade algorithm [68], which has previously been shown to be more efficient and accurate in extracting respiration-related movements if compared to other dense methods [23].

## 7. Lameness and Lying Behavior

Lameness is one of the most widespread health problems among dairy cattle since it causes pain, gait alterations, and negatively affects reproduction and milk production, leading to economic losses for the farmers [54,69]. This problematic issue is strictly related to the barn management: incorrectly balanced feed, unclean bedding material, and structures that do not prevent heat stress may cause a rise in pathological cases [70]. However, scoring lameness performed by trained observers is a time-consuming activity that is not feasible in large-scale farming contexts [71]. Therefore, the development of instruments that provide an early detection of lameness for the individual cow is of great importance to protect the animal’s welfare and the farm’s economy. Accelerometers have been proposed to monitor cattle, but, as wearable sensors mostly applied to the legs, they can cause discomfort and stress. Recently, computer vision has been proposed to automate the lameness scoring process and to improve early detection in large herds. To accomplish this objective, CNNs (convolutional neural networks) have been applied for object recognition and RNNs (recurrent neural networks) for feature extraction, allowing for finding a correlation between the lameness gravity and the degree of curvature of the cows’ back [72,73]. Machine learning techniques allow researchers to focus on leg swings and movement patterns to classify lame cows [74] based on the extraction of features related to the movement characteristics, such as symmetry and stride length [20]. Nevertheless, considering a singular characteristic as a basis for lameness detection could lead to false positives or inaccuracies, in particular if the cows are not severely lame [73,75]. Lame animals, in fact, show different gait characteristics, such as greater arching and flattening of the back and an increase in head movement while moving, to minimize the stress on the lame leg that would lead the cow to feel more pain [76,77]. The results obtained underlined that Mask R-CNN shows an excellent lameness-detection accuracy (98%) but still has some issues in locating dark feet and legs if the background is dark due to the low contrast between the objects. Ref. [20] proposes a novel approach that involves the identification of three keypoints on the back of the animal that define the back’s curvature and allow the calculation of the variance in movement and the degree of lameness. In the study, different machine learning algorithms were tested for their efficacy, showing AdaBoost as the most accurate algorithm with an overall accuracy of 77.9%. Another study instead proposed a method that enables recognition and analysis of both posture and gait at once, leveraging a Mask R-CNN to estimate different keypoints on the cows’ body to determine back arching and head position. Subsequently, the CatBoost algorithm selects useful and nonessential features to correctly classify each cow for its lameness degree. This study showed evidence that the rear of the animal could provide more information than the front, in accordance with the fact that the majority of causes of lameness affect the hind legs [77]. It is important to notice that irrelevant information extracted from videos can reduce the accuracy of lameness detection: in this regard, a Dimension-Reduced Spatiotemporal Network (DRSN) has been proposed by [78] to manage irrelevant information and reduce its impact on the overall machine learning algorithm performance. The location of the hooves provided with YOLOv4 has been used as a base to obtain a spatiotemporal image from the video; accordingly, the DenseNet algorithm performed the lameness classification on the spatiotemporal image, receiving as output the locomotion score of the cow. This method obtained 98.50% accuracy for the classification performed by DenseNet, proving that this method could effectively reduce the inaccuracies leveraging a DRSN [78]. To enable individual lameness classification using computer vision and deep learning, cameras should be placed in areas where all animals transit daily. Therefore, the entrance or exit of the automatic milking system or milking parlour represents the ideal location for these devices. This camera positioning can create some problems related to the fact that along the walkway that leads inside or outside the milking area, the cows follow one another very quickly: in this regard [79] stated that the time interval between following animals must be at least 35 s; otherwise, the video recording and analysis of the video frame will not give an accurate output.

Detection of the herd movement pattern can also provide information on the lying behavior of the cows, which is a strong indicator of the level of comfort and physical health of the herd and indirectly of the suitability of the cubicles [80]. Both welfare assessment programs and farm managers use this parameter on a daily basis to assess animal welfare and comfort, but traditionally the observations have been visually carried out [22], which found no application in the context of large livestock farming as a time-consuming and subjective activity.

Recent progress of computer vision technology makes it the right tool to obtain the precise computation of the animal’s biomechanisms and to monitor the onset of altered kinematic behaviours during posture transitions. Another parameter that can be monitored to assess animal welfare and health is lying time [24]: for instance, cows that are suffering from heat stress increase the time spent standing, negatively affecting rumination, hoof health, and milk yield [44]. Ref. [22] proposed a 3D pose estimation software able to compute the variation between sacrum height and withers height and therefore infer the lying-to-standing behavior, obtaining a sensitivity of posture transition detection of 88.2%.

## 8. Mastitis and Milk Yield

Mastitis is a widely spread and complex disease which leads to great economic loss and causes alterations in milk quality and yield. Nowadays the pressure on the livestock sector to reduce the antimicrobial drugs is increasing due to the increasingly diffuse antimicrobial resistance; since the treatment for mastitis is the most common reason for the use of antimicrobial drugs on dairy farms, its control is of critical importance [81]. Early detection of health issues related to the udder is essential for the farmer to intervene on cow feeding management or stall cleaning, with the aim of preventing a mastitis onset [82]. It has been taken into consideration that clinical and subclinical mastitis do not present the same symptoms: the first presents phenotypic alterations that are usually visible to the naked eye, while the second is identifiable by more subtle alterations that are not visible [83]. The most common diagnostic methods for both clinical and subclinical mastitis include analysis carried out on milk, but those methods require expensive instruments, pre-treatment processes, and qualified personnel to carry out the analysis, resulting in extended detection periods [13]. Infrared Thermography (IRT) represents an alternative, non-contact method [84] through which the udder’s health could be continuously monitored and in real-time, identifying any increases in skin temperature, which indicate the presence of inflammation [85].

IRT is considered an efficient instrument to carry on this kind of measurement, especially when combined with deep learning algorithms that enable the detection of keypoints on the dairy cow udders reported in the thermal images in order to relate temperature and size features and obtain an accurate mastitis detection [13,86].

In this regard, authors of [19] propose to use YOLOv5 for detection and recognition of mastitis, based on the comparison of eye and udder temperatures: The setting of a proper threshold for the temperature difference between these two points has been crucial to performing an accurate detection. The threshold for the temperature difference between udder and eye has been set at 0.8 °C, while the threshold between the left and right sides of the udder has been set at 0.72 °C. Leveraging this method, the authors obtained a mastitis detection accuracy of 85.71%. It must be considered that skin surface temperature can be easily influenced by environmental parameters and that the data recorded for this parameter can be affected by the cleanliness of the skin [13]. Ref. [13] underlines that detection methods based on only one indicator can be limited and show less accurate performances; instead, they propose an alternative method, combining the use of YOLOv7 with the CenterNet deep learning algorithm. The authors obtained a higher accuracy in detecting and predicting the degree of mastitis (88.71%) by performing detection and prediction of dairy cow mastitis leveraging two indicators: size and temperature features of the udder, obtained by thermal images. The morphology and size of the dairy cows’ udders have been identified as useful factors for prediction and evaluation of both subclinical and clinical mastitis at their primal stages. Previous research showed that animals that present specific udder attachments, heights, and widths were correlated with a greater risk of mastitis [82,87]. Leveraging thermal images, the temperature difference between the background and the animal can be used to analyse both morphologic and temperature features. Milk yield is another important aspect considered by farmers when evaluating udder health in order to consciously perform many management choices on reproduction, such as drying off and heat detection [83]. The evaluation of this parameter is carried out mostly at milking time, in particular by automatic milking systems [86]; nevertheless, many barns nowadays still adopt traditional milking systems that are more unlikely to provide individual information related to the milk yield of the cows [88].

Computer vision has found an application in this regard as a non-contact technology that can be combined with multiple sensors for several applications. Ref. [89] proposes a lower cost alternative compared to traditional milk yield estimation methods, that is, the application of an infrared camera to obtain 3D space thermal images of the udder before and after milking, and thanks to a specific algorithm, the volume of the udder could be computed before and after the milking process. Although this method may have some limitations in accuracy, it offers a low-cost and original approach that could be applied in future research.

## 9. Social Behavior and Oestrus Detection

As highly social animals, bovines have evolved to live in groups that are regulated by social hierarchy and different levels of association with conspecifics [90]. However, typical dairy barn management practices provide almost continuous regrouping of the animals. Regrouping, together with limited available space to perform social interactions, causes an adverse effect on cows’ welfare and productivity [91]. Current methods to monitor cows’ behavior are either wearable sensors, whose measurements and accuracy can be negatively impacted by losses or damage, or time-consuming, as they rely on trained observers to evaluate the recorded videos of the cows [92]. Additionally, the great amount of data generated by sensors employed in large-scale farms increases the need for greater storage systems that are not always easily available for the farmers [93].

To better understand social behaviours and relationships, computer vision represents a promising and non-invasive method to analyse this topic and evaluate how the structures and the devices present in the farm are used by the animals, such as automatic milking systems [94,95] or brushes [96]. Ref. [95] positioned the cameras to have a focus on automatic milking systems and leveraged EfficienDet combined with a Euclidean-based tracking model in order to work on spatial proximity between couples of animals; the CNN-based detector allowed the recognition of each animal, and the tracking model allowed the tracking of temporal variations in the interactions between the animals in terms of space and time.

The analysis of time as an essential factor to track the various behaviours and interactions during day and night periods has to take into consideration the variation in light intensity in the background that increases the difficulty in the image elaboration and evaluation [97]. To overcome this issue, Ref. [98] proposed a method that takes into consideration different weather conditions, leveraging RGB channel synthesis technology to simulate, for instance, different light intensities, and with this implementation, they managed to obtain an average accuracy of 90.2% in the recognition of basic behaviours (i.e., eating, standing, lying, walking), and that could be further developed to recognize social interactions between cows. Moreover, a crucial aspect to consider in behavior analysis is the type of interaction that the animals enact, which can be either affiliative (positive) or agonistic (negative), of which the latter usually indicates the presence of welfare issues.

Usually, cameras that focus on behavior classification are positioned in specific areas of the barn in which aggressive behaviours are more likely to be found due to the competition that rises between cows that are in different hierarchical positions for access to feed, milking, or spaces. Feeding and waiting areas are places where these behaviours typically occur [99,100]. In this regard, wearable devices can detect social interactions based on spatial proximity but are not able to identify and classify the behavior itself. Therefore, a system, for instance, such as Ultra-Wide Band (UWB), could be implemented together with a CNN so that the first can be used to track the cows and the second to classify the interactions between them [99]. An alternative method to classify cow interactions is given by [100], which computes distances as geometric features and has investigated the alignment of the cows’ shapes, obtaining, however, an accuracy of nearly 85%. Accuracies obtained in the studies that focused on the recognition and classification of animals’ interactions remained under 94%, proving the difficulty of applying computer vision methods in this field and for this objective. The reaction to a stimulus of an animal is due to the external factors that created the stimulus but also on the individual experience and characteristics; hence, to recognize a single movement, the algorithms employed have to consider a range of inter- and intra-class variability, representing a potential issue to resolve in order to improve these technologies [97]. Further investigation is needed regarding the social interactions and, more generally, the study of animal behavior, since it is a fundamental prerequisite for the maintenance and optimization of health, welfare, and production of dairy cattle barns.

Another aspect that can be monitored through image processing and that could give us information on the physiological state of the animals is oestrus. A well-structured reproduction management strategy is crucial to ensure livestock welfare and productivity. Incorrect oestrus identifications lead to a decrease in the reproductive efficiency of the cows [101,102]. Various methodologies have been employed to detect cows in heat, such as devices attached to the tail that utilize chalk or dye; accelerometers [103] attached to collars or pedometers [104]; and thermometers that monitor the internal temperature changes associated with oestrus manifestation [105].

These technologies are wearable or need direct contact with the animal to record data, causing them to be potentially stressful for the cows, and additionally, the devices that measure activity and internal temperature rely on indirect indicators of heat; hence, they could provide false positives caused by physiological alterations different from oestrus [106]. One of the most relevant signs of oestrus is the passive reaction of a female cow to other cows mounting her [101], and this action can be detected through a machine-vision approach. Ref. [106] considered as a threshold to classify a cow as in oestrus 2 s of following behavior that evolves into 2 other seconds of mounting behavior. The authors underline the need for an implementation of the number of indicators or thresholds to help the algorithms discern false from true oestrus events.

## 10. Conclusions and Future Research Directions

This literature review has examined the implementation of deep learning models in computer vision and machine learning techniques, focusing on the applications related to the management of dairy cow barns, highlighting the practical impact of such innovations in everyday farming operations.

The main conclusions of this review paper can be summarized in the following three points:

1. Compared to traditional wearable devices, computer vision provides a stress-free, cost-effective, and scalable method for monitoring dairy cattle. By eliminating the risk of equipment loss or damage and avoiding direct contact with animals, it ensures continuous data collection while safeguarding animal welfare and reducing economic losses for farmers.
2. The integration of advanced models such as CNNs and YOLO-based architectures has transformed what is possible in livestock monitoring. These methods enable precise, real-time detection of subtle physiological and behavioural changes, allowing earlier identification of health and welfare problems. In turn, this supports more accurate decision-making, targeted interventions, and improved productivity in increasingly large-scale dairy operations.
3. Despite remarkable advances, challenges remain in ensuring that algorithms are robust across diverse farm environments, lighting conditions, and growth stages. To unlock the full potential of computer vision, future research must focus on building larger and more heterogeneous datasets, integrating multimodal data sources, and validating systems under real-world conditions. These steps are crucial to make computer vision a cornerstone of sustainable, welfare-oriented dairy farming.

The dairy sector faces growing challenges due to climate change and the technological transformation of modern livestock systems. Within this context, the present review may contribute to enhancing both welfare conditions and productivity in dairy farming.

## Figures and Tables

**Table 1:** Details from the papers related to the monitoring of BCS in dairy cattle.

Monitored Feature	Reference	Details on the Camera Adopted
BCS	[9]	Kinect V2 sensor (Microsoft; Redmond, WA, USA) Intel^®^ RealSense™ Depth Camera D435 (Intel^®^ Corporation, Santa Clara, CA, USA)
	[56]	Intel^®^ RealSense™ Depth Camera D435 (Intel^®^ Corporation, Santa Clara, CA, USA)
	[57]	Microsoft Kinect (Microsoft; Redmond, WA, USA)
	[58]	Microsoft Kinect (Microsoft; Redmond, WA, USA)

## Data Availability

No new data were created or analyzed in this study. Data sharing is not applicable to this article.
